# Treatment and Resolution of an Implant Periapical Lesion (IPL) with Guided Bone Regeneration

**DOI:** 10.1155/2023/6624249

**Published:** 2023-10-12

**Authors:** Carlo Sgarbanti, Frank Armando Mauro

**Affiliations:** ^1^Department of Dental Diagnostic and Surgical Sciences, Dr Gerald Niznick College of Dentistry, Rady Faculty of Health Sciences, University of Manitoba, Winnipeg, MB, Canada; ^2^Thunder Bay Regional Health Sciences Centre, Thunder Bay, ON, Canada

## Abstract

**Background:**

Implant periapical lesion (IPL) is a rare condition that can affect dental implants. Several different approaches have been proposed for the treatment of this condition. Awareness and literature discussing this condition and possible treatment options have grown significantly in the last 25 years. *Case Presentation.* The present case report describes the treatment of an implant periapical lesion with a combined approach consisting of surgical lesion removal, mechanical instrumentation with titanium brush, detoxification with tetracycline, and guided bone regeneration (GBR) with demineralized allograft bone and cross-linked collagen membrane. The patient was followed up for 6 months postoperatively, showing complete resolution of the buccal fistula. No signs or symptoms of discomfort or pathology were reported.

**Conclusions:**

The case report presented a combined approach that can be successful in the surgical treatment of an IPL in which the implant stability is maintained.

## 1. Introduction

Implant periapical lesion (IPL) also known as apical peri-implantitis or retrograde peri-implantitis is a rare condition with an incidence ranging between 0.26% according to Reiser and Nevins [1] and 1.6% in the maxilla and 2.7% in the mandible, according to Quiryen [2]. A recent retrospective study [3] reported an incidence of IPL of 3.7%. In that study, the incidence increased in cases with adjacent teeth that had periapical radiolucency. IPLs were described for the first time by McAllister in 1992 [4] and since then have been reported in several case reports and case series. Different possible etiologies have been proposed, among them residual bacteria in the implant site area or endodontic lesions on adjacent teeth, which have been reported as the most frequent [5]. Other possible etiologies are overheating during implant preparation, violation of minimal distance from adjacent teeth, overpreparation in length of the surgical site, bone compression, fenestration of the buccal bone, peri-implantitis, implant fracture, premature loading, implant surface contamination, bone grafting procedures, cigarette smoking, reverse torque test, overtorquing, parafunctional habits, operator experience, and systemic factors [5–7].

A retrospective study conducted by Burdurlu et al. [8] found no correlation between demographic factors and IPL, a limited correlation with implant-related factors such as implant brand, surface, and size, and a strong correlation between surgical technique, local bone factors, reasons for tooth loss, and the condition of the adjacent teeth. No definitive consensus exists about how to classify IPLs [9]. Classifications have been proposed by Sussman in 1998 [10] and more recently by Sarmast et al. [11].

Other classifications proposed by Reiser and Nevins [1] and by Peñarrocha-Diago et al. [12] are based on the clinical presentation of this condition. The purpose of the present case report was to describe the diagnosis and the management of an IPL and the resolution with a combined approach including guided bone regeneration (GBR).

## 2. Case Presentation

**Figure 1:**
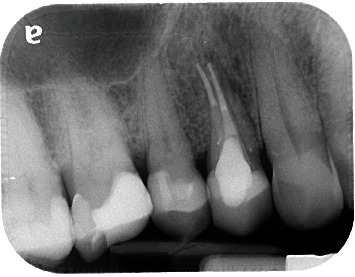
Radiograph of the upper right first premolar showing radicular radiolucency.

**Figure 2:**
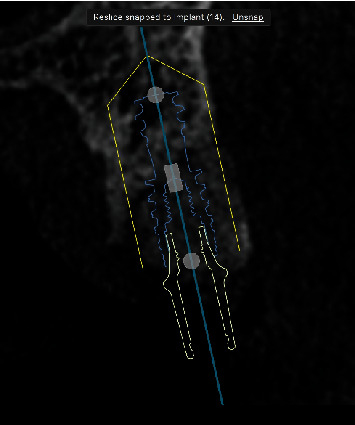
CBCT scan showing adequate bone volume to proceed with the implant placement.

**Figure 3:**
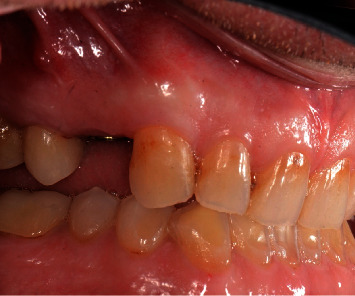
Clinical presentation of a fistula in the buccal mucosa apical to the implant.

**Figure 4:**
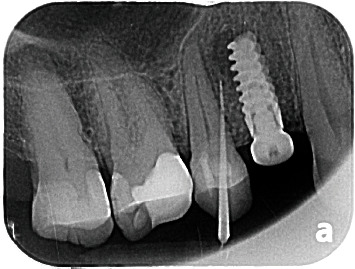
Radiographic presentation of the implant periapical lesion.

A 60-year-old male, ASA I patient, without previous history or treatment of periodontal disease was referred to a private periodontal office for extraction and replacement of an upper right first premolar with history of endodontic treatment and restorative hopeless prognosis due to vertical root fracture (Figure 1). Extraction and ridge preservation with mineralized allograft bone (Puros Cancellous Particulate Allograft, O.5 cc, 250-100 *μ*m, Zimmer Biomet) and cross-linked collagen membrane (Socket Repair Membrane, 10 × 20 mm, Zimmer Biomet) were performed. Four months postoperatively, a CBCT scan of the area was taken showing adequate bone volume to proceed with the implant placement (Figure 2). The patient accepted and signed the treatment plan. A regular platform tapered implant (NobelActive 4.3 × 11.5 mm, Nobel Biocare USA, LLC) was placed, primary stability was achieved, and a healing abutment was placed. The healing was uneventful, and the patient did not report any discomfort. Restorative procedures were delayed by several months due to the patient's personal commitments and the COVID-19 provincial lockdown. Fourteen months later, the patient returned to the office for an implant follow-up appointment prior to proceed with the restorative treatment plan. The implant presented stable; however, the patient reported slight discomfort when pressing on the buccal mucosa apical to the implant, and minor swelling over the implant was noticed. He reported that some discomfort started 2-3 months after the implant placement. Clinically, the patient had developed a fistula in the buccal mucosa apical to the implant and to the second upper premolar (Figure 3). The area was painful upon palpation. A periapical radiograph was taken (Figure 4) showing a periapical lesion apical to the implant but also proximal to the apex of the distal neighboring tooth. Probing depth around the implant was within normal limits without bleeding on probing (BOP) or suppuration. Due to these clinical and radiographic findings, the restorative treatment of the implant was postponed.

Once the buccal fistula was noticed, a systemic antibiotic therapy with amoxicillin 500 mg TID for 7 days was prescribed without achieving resolution of the fistula. The patient was referred back to his restorative dentist to assess the vitality of the upper right second premolar which proved to be nonvital.

**Figure 5:**
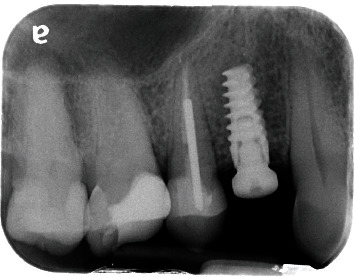
Endodontic treatment of the upper right second premolar.

**Figure 6:**
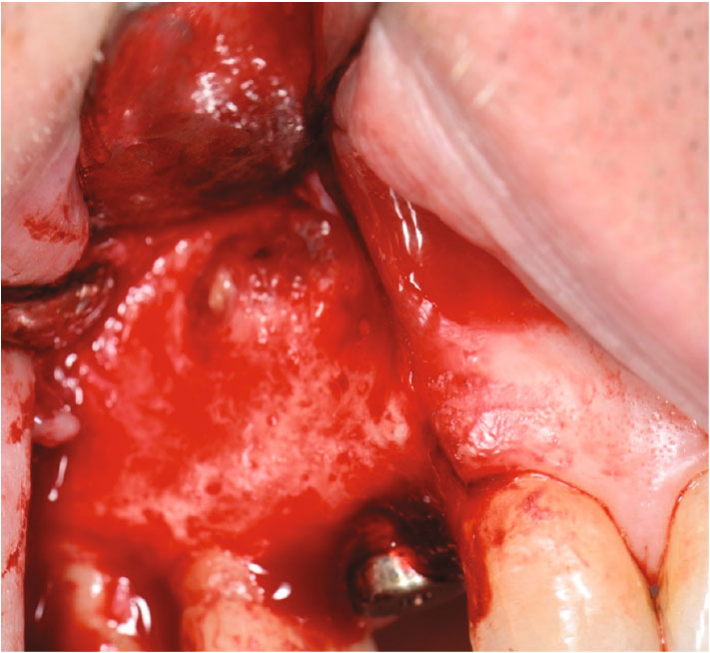
Full-thickness flap reflected showing granulation tissue around the implant apex.

**Figure 7:**
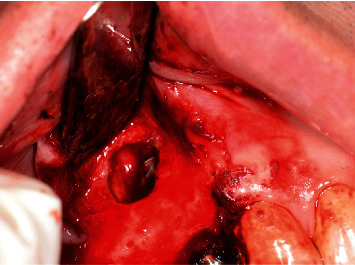
Granultion tissue completely removed.

**Figure 8:**
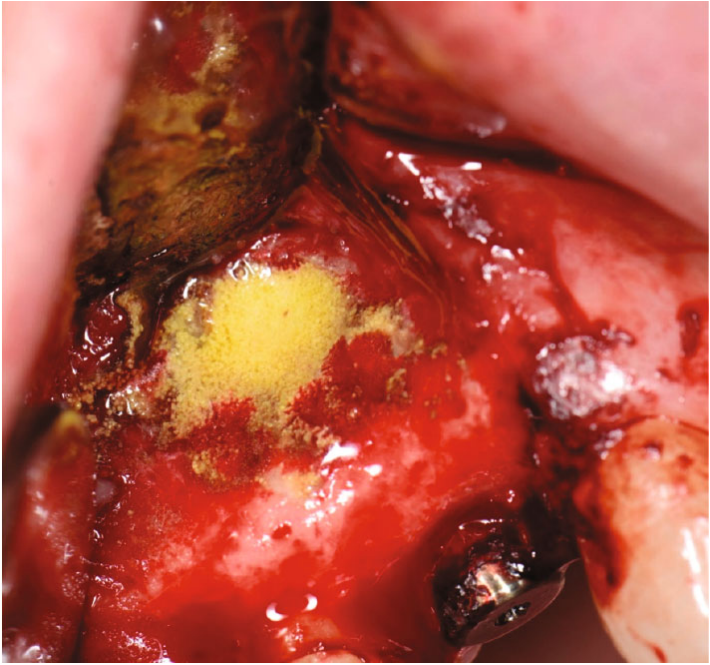
Decontamination with tetracycline paste for 2 minutes.

**Figure 9:**
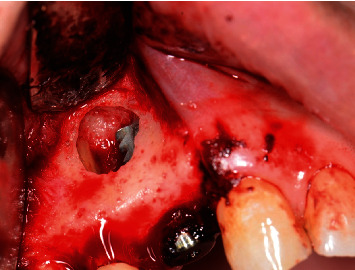
Presentation of the exposed implant treads after decontamination with tetracycline.

**Figure 10:**
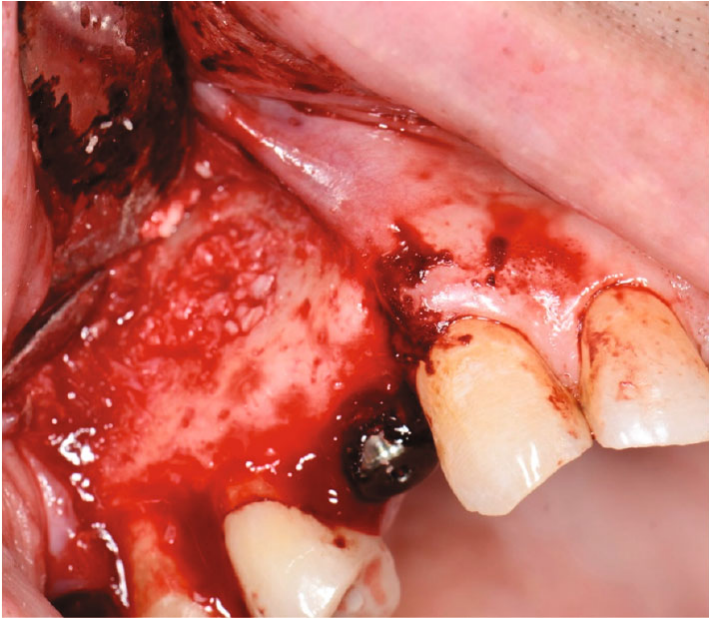
Demineralized ground cortical bone placed around the apex of the implant.

**Figure 11:**
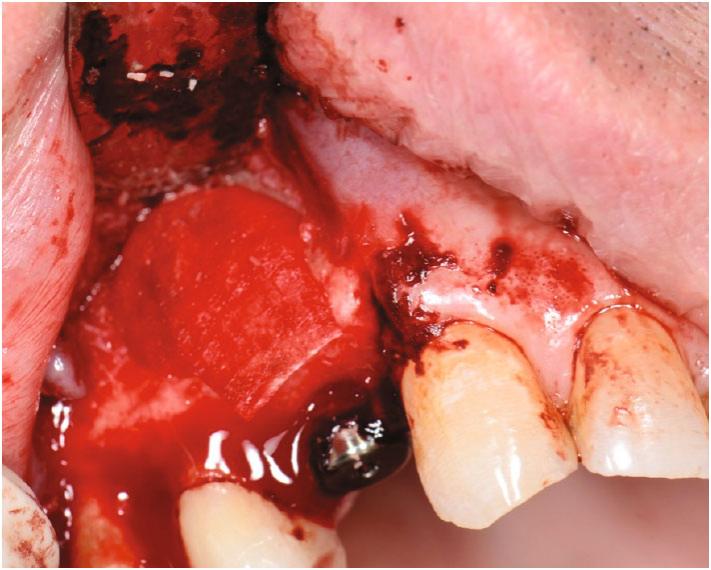
Collagen membrane placed over the grafted site.

Endodontic treatment of the nonvital tooth was completed (Figure 5), and two months of healing time were allotted prior to follow-up. The fistula remained, and the patient was informed that surgery was needed to address the lesion. The patient accepted and signed the treatment plan and understood the risks and the benefits of the surgery, and signed the consent form. Implant stability was assessed, and the implant was countertorqued at 35 Ncm without signs of movement or discomfort for the patient. Local anesthesia was performed on the buccal and lingual aspects from the right upper canine to the right first upper molar with 1.5 carpules of 2% lidocaine with 1 : 100.000 epinephrine, by means of local infiltrations. Using a 15c blade, a crestal full-thickness incision from the distal aspect of the right upper canine to the mesial aspect of the right first upper molar was performed along with 2 vertical incisions distal to the canine and mesial to the first molar to better access the IPL. A full-thickness flap was then reflected showing an area at the apex of the implant in which the cortical bone was completely resorbed and replaced by granulation tissue (Figure 6). Using spoon curettes, the granulation tissue was carefully removed (Figure 7). Foreign body fragments consistent with endodontic material incapsulated in the granulation tissue were noticed. Implant treads were then carefully brushed using a titanium brush (Straumann TiBrush, Straumann Group) [13] with an oscillating low speed (<900 rpm) and irrigation. The bone defect presented clinically with a width of 8 × 8 mm and depth of 9 mm. Almost 50% of implant bone loss was observed. The area was then decontaminated using a tetracycline paste created mixing 250 mg of tetracycline powder with sterile saline solution (Figure 8). The tetracycline paste was left in place for 2 minutes and then was carefully rinsed with saline solution (Figure 9). The bone defect was filled with 0.5 cc of allograft particulate bone graft (AlloGraft demineralized ground cortical 250-1000 *μ*m, Straumann Group) (Figure 10) covered with a cross-linked collagen membrane (BioMend Collagen Membrane, Zimmer Biomet) 15 × 20 mm (Figure 11).

**Figure 12:**
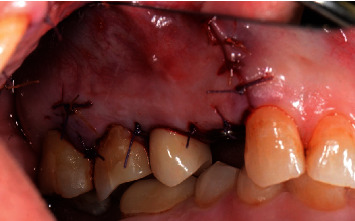
Surgical site sutured with primary closure.

DFDBA bone was chosen for its ability to attain new bone formation [14] and for successfully resolution of similar cases [4, 15]. The flap was sutured with single interrupted Vicryl 5-0 and chromic gut 5-0 sutures, and primary closure was achieved (Figure 12). The patient tolerated the procedure very well. postoperative instructions and prescriptions were provided (amoxicillin 500 mg TID for 7 days and ibuprofen 400 mg 3 times a day for 3 days). The patient was also instructed to rinse with chlorhexidine 0.12% twice a day until suture removal. All patient's questions were answered promptly.

**Figure 13:**
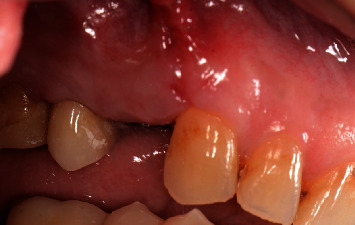
Sutures removed at 10 days postoperative appointment.

**Figure 14:**
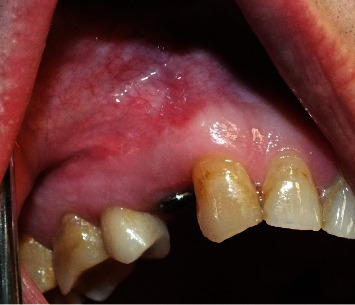
Complete clinical healing at the 6-month postoperative appointment.

**Figure 15:**
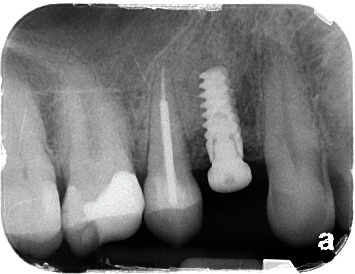
Radiographic healing, showing normal bone density at the 6-month postoperative appointment.

The patient returned for a 10-day post-operative follow-up appointment and suture removal appointment (Figure 13). The patient reported limited postoperative discomfort and complete resolution of the buccal fistula was noticed. The patient then returned for a 6-month follow-up appointment in which a new periapical X-ray was taken (Figures 14 and 15) showing normal bone density around the implant and there were no clinical signs of a residual IPL.

## 3. Discussion

Limited data is available regarding IPL and is mostly based on case reports and case series. A definitive treatment protocol for IPLs has not been established. Several different approaches have been proposed [9]: implant removal, surgical excision of the periapical lesion with or without biomaterials [16], decontamination with air-abrasive device and laser [17], and also implant apicectomy [18, 19]. A decision tree has been proposed by Sarmast et al. [7] to guide the clinician in choosing the appropriate treatment plan for symptomatic and asymptomatic implants with IPLs. This case has been successfully treated using a combined approach consisting of surgical lesion removal, mechanical instrumentation with titanium brush, detoxification with tetracycline, allograft and collagen membrane. The literature reports that particular attention should be given to the first 3 months after implant placement to early diagnose this condition [9]. Even the presented case may have had an early symptomatic presentation, but unfortunately, due to specific circumstances such as the patient's personal commitments and provincial COVID-19 lockdown, a definitive diagnosis was delayed. Peñarrocha-Diago et al. differentiated between acute nonsuppurated IPLs, acute suppurated IPLs, and subacute IPLs [20]. In the acute stage, there is a well-localized acute pain at the implant apex. The nonsuppurated acute stage presents no radiographic signs; the suppurated acute stage presents with periapical radiolucency. In the subacute stage, the symptoms are usually very mild, and often there is a buccal fistula and a periapical radiolucency. According to this classification, the case presented in this case report should be classified as subacute due to the mild discomfort reported by the patient and the presence of fistulous tract and radiographic radiolucency. Peñarrocha-Diago [20] also proposed a decision-making chart, according to this chart subacute, cases should be differentiated in cases with implant mobility and in cases without implant mobility. Where there has been a loss of implant stability, the implant should be removed; in cases of maintained implant stability, periapical surgery should be considered. This case report adds to the literature a case of an IPL successfully treated in which the implant stability is maintained.

## 4. Conclusions

The present case report showed a combined approach for the surgical treatment of a large IPL.

## Data Availability

The data used for the current study are available from the corresponding author upon request.
